# Effectiveness of message-framing to improve oral health behaviors and dental plaque among pregnant women

**DOI:** 10.1186/s13690-021-00640-1

**Published:** 2021-06-28

**Authors:** Masoumeh Divdar, Marzieh Araban, Akbar Babaei Heydarabadi, Bahman Cheraghian, L. A. R. Stein

**Affiliations:** 1 Department of Health Education and Health Promotion, School of Health, Ahvaz Jundishapur University of Medical Sciences, Ahvaz, Iran; 2 Social determinants of health research center, Department of Health Education and Promotion, Public Health School, Ahvaz Jundishapur University of Medical Sciences, Ahvaz, Iran; 3 Department of Biostatistics and Epidemiology, Ahvaz Jundishapur University of Medical Sciences, Ahvaz, Iran; 4 Department of Psychology, University of RI, Kingston, RI USA; 5 Social/ Behavioral Sciences and Center for Alc/ Addic Studies, Brown University School of Public Health, Providence, RI USA; 6 Department of Behavioral Healthcare, Developmental Disabilities & Hospitals, Cranston, RI USA

**Keywords:** Oral health, Pregnancy, Message-framing, Dental plaque

## Abstract

**Background:**

Oral health is considered a prominent factor that contributes to quality of life. Hormonal changes during pregnancy can influence oral health. Message framing can play an important role in oral health. The aim of the present study was to investigate the effect of message framing on oral health and dental plaque among pregnant women.

**Methods:**

The study was conducted in 2017 on 108 pregnant women in Izeh county, Iran. Participants were randomly assigned to gain-framed, loss-framed, and control groups. The research instrument included a two part questionnaire containing demographic information and oral health knowledge, attitude, behavioral intention, self-efficacy, practice, and dental plaque index. Gain-and loss-framed messages were sent to the intervention groups via cell phone texts, but the control group did not receive any messages. Participant dental plaque was clinically assessed. Analysis of covariance with follow-up tests were performed using SPSS version, 23.0 with *p*-value set at 0.01 for significance.

**Results:**

Intervention groups had better oral health (knowledge, atttitude, intention, efficacy, practices and plaque) scores compared to the control group (*p* < 0.001), but intervention (gain- vs loss-framed) groups did not differ on outcomes.

**Conclusion:**

Text message intervention improved knowledge, attitude, behavioral intention, self-efficacy, practice, and dental plaque among pregnant women. While differences between control and both intervention groups indicated text messaging had an impact on oral health outcomes, message framing (i.e., gain vs loss) had no discernable impact on oral health outcomes.

**Supplementary Information:**

The online version contains supplementary material available at 10.1186/s13690-021-00640-1.

## Background

According to the World Health Organization (WHO), *oral health* is a prominent indicator of *overall health*, well-being and quality of *life* [1]. Moreover, oral health is considered as an important factor that determines different aspects of quality of life (physical, mental, and socioeconomic) [1, 2]. The burden of oral diseases has also grown by 20% from 1990 to 2010 globally [3]. In particular, oral health of persons in Iran can be categorized as only moderate according to a WHO report from 2000 [2]. Many adults world-wide are at risk of periodontal disease and cavities [4, 5] caused by bacterial activity in dental plaque [6]. To improve oral health, prevention programs need to be performed before birth [7]. Hormonal changes and nutritional conditions can make pregnant women susceptible to gum disease and cavities [1, 8, 9]. Morning sickness with vomiting, over-consumption of sugar, and less oral hygience (e.g, tooth brushing, flossing) can lead to formation of microbial plaque and cavities [10]. Periodontal diseas is also associated with antenatal and natal complications such as early birth, low birth weight, limited intrauterine growth, or reduced embryo size given gestational age [8, 9, 11, 12]. As compared to non-pregnant women, pregnant women are at increased risk for cavities [13]. Pregnant women use less dental care and ignore oral hygiene as compared to the general population. World-wide, 58–65% of pregnant women are not committed to oral care [1, 14, 15]. In Hamadan, Iran, only 68% of pregnant women brushed their teeth once a day, and only 11.8% did so after each meal [10]. Pregnant women report several factors hindering oral care including lack of information, insufficient time, and fear of dental treatments [16]. Oral health education may be a critical factor in preventing plaque formation and dental disease [17]. However, the effectiveness of a health education program depends largely on the use of an appropriate educational theory [18].

Tversky & Kahneman (1981) [19] proposed that health messages can be framed in terms of either the benefits of engaging in the recommended behaviour (gain-framed messages) or the costs of not engaging in the behaviour (loss-framed messages). Although conveying essentially identical information, one form of message-framing may be more effective at promoting health behaviour change than the other. Specifically, loss-framed messages might be persuasive for illness detection behaviours, such as X-ray for cavity detection, while the gain-framed messages should be more persuasive for illness prevention behaviours, such as tooth-brushing to promote oral health [20].

Findings have been mixed with respect to effects of gain- or loss-framed messages. One study showd no differences in gain- or loss-framed messages across domains [21]. However, Updegraff et al. (2015) showed that participants who watched a video where the frame (gain/loss) matched perceived susceptibility (low/high) had significantly greater likelihood of flossing [22]. Ramezankhani et al. (2016) showed that the attitude, intention and behavior of flossing and tooth-brushing increased in students who received a gain-framed message compared to those who received loss-framed message [23].

A study in United States revealed a large percentage of adults (92%) owned a cell phone, allowing distribution of health information through text messages [21]. Therefore, although a comprehensive review [24] found that effects of framing may be small, given the ease of distributing such messages via cell-phones, texting may be an effective means to impact oral health on a wide scale, among persons particularly vulnerable to oral health problems. Accordingly, the objective of this study was to investigate the effectiveness of message-framing on oral health-related behaviors and dental plaque among pregnant women. The study had two hypothesis: 1) Message-framing intervention will improve oral health behavior and plaque in comparision to control condition (no message); and 2) Pregnant women receiving a gain-framed intervention will improve significantly more than pregnant women receiving loss-framed intervention.

## Methods

### Participants and randomization

The study took place in Izeh county of Iran from November 2017 to February 2018. Potential participants were pregnant women referred to a birth and counseling center for antenatal training classes. Following screening and recruitment, *N* = 108 participants were randomly assigned to intervention (receive either gain- or loss-framed text messages) and control (did not receive any messages) groups using block randomization [25] Block randomization reduces bias and achieves balance in the allocation of participants to interventions, especially with small sample size. This method increases the probability that each arm will contain an equal number of individuals by sequencing participant assignment by block.

### Inclusion criteria

The inclusion criteria were as follows: Abilty to read and write; access to cell phones; willingness to participate; not having a high risk pregnancy according to mid-wife; not having an underlying disease (e.g., cardiovascular disease, autoimmune disease, cancer, diabetes, etc.); gestational age of 16 to 28 weeks; and being between 18 to 35 years, inclusive. Participants were selected using non-probability convenience sampling.

### Intervention

Two cell phone numbers were obtained from pregnant women for delivey of messages and follow-up. Prospect Theory [24] indicates that persons respond differently to messages depending on whether they are framed in terms of benefits (gain) or costs (loss). Messages (*n* = 30) had similar content, but were framed in terms of advantages/ disadvantages of using/ not using dental hygiene (e.g., tooth-brushing, flossing, using mouth-wash, etc). Message design was based on this research group’s prior work in gain−/ loss-message framing [26]. Gain- and loss-framed messages were sent once per day based on group assignment, whereas women assigned to the control condition received no messages. The gain-framed group recieved message such as, “If you floss every day, you will have a beautiful smile,” whereas the loss-framed group received messages such as, “If you do not floss every day, you may be embarrassed with your smile” [27]. Women were asked to briefly reply that the messages had been received. If researchers did not receive this confirmation within 3 days [28], a member of the research team would call, or send educational messages through the other cell phone number. One unique text was sent per day to each intervention group for 30 days.

### Outcomes measures

All measures were collected in person prior to intervention. Eight weeks after sending the messages, the post-test questionnaire was completed by the three study groups in person.

#### Demographic/ pregnancy information

Demographic data and data on pregnancy history were collected: Maternal age, husband’s age, duration of marriage, gestational age, maternal occupation, husband’s occupation, insurance information, maternal education, husband’s education, previous pregnancy, maternal ethnicity, household income, family size, and place of residence.

#### Knowledge

Knowledge of how to prevent oral health problems was assessed via 14 items with correct answers = 1 and incorrect answers = 0; range could be 0–14. Oral health attitude (10 items; total score range = 10–50), behavioral intention (6 items; total score range = 6–30), and self-efficacy (9 items; total score range = 9–45) were rated on a 5-point Likert scale from 1 = Totally Disagree to 5 = Totally Agree (3 = Neutral). After reverse scoring, higher scores were indicative of higher levels of each construct. Oral health practice (14 items; total score range = 0–14) were scored as 1 = correct response and 0 = incorrect response. Direct observation was used to score some items with demonstration on a model (tooth brush at 45^°^ for different parts of the tooth, vibrating movements on gum lines, horizontal movement on occlusal surface, vertical movement on anterior and internal surfaces, use of suitable floss size, correct winding of floss around fingers, correct movement of floss between the teeth and gum line, etc.). Higher practice scores reflect better oral health practices.

##### Validity and Reliability

Content validity and face validity were evaluated. Opinions of 10 experts were elicited. After summarizing expert opinions, the content validity ratio (CVR) and content validity index (CVI) were calculated based in scientific recommendations [29] and found to be CVR = 0.99 and CVI = 1, both excellent scores [30]. To measure face validity, 10 pregnant women were asked to rate the importance of each item on a 5-point Likert scale (5 = absolutely important, important, moderately important, slightly important, and 1 = absolutely not important). The importance rating for the questionnaire was Mean (M) = 4.5, indicating acceptable face validity overall.

A separate sample of woment meeting inclusion criteria (*n* = 10) completed the questionnaire once and then again 2 weeks later, producing the following test-retest correlations for attitude, intention, efficacy and practice, respectively: r = 0.89, 0.89, 0.91, 1.0. Knowledge produced Cronbach alpha of α = 0.80.

#### Oral health examination

A dentist examined the women’s teeth and obtained the percentage of dental plaque using the Naval Plaque Index (NPI [14, 31]). Scores can range from 0to 100%, with higher scores reflecting more plaque.

### Sample size

To determine sample size, α was set to 0.01 and β to 0.1 with effect sizes based on similar prior work [5]. In particular, the below equation was used where *x*_1_ = 0.07 (gain-frame flossing mean change), *x*_2_ = 0.09 (control group flossing mean change), *s*_1_ = 0.147, and *s*_2_ = 0.123. Given this, a total of *N* = 108 participants were estimated in 3 groups of 36 per group.

$$ n=\frac{{\left({S}_1^2+{S}_2^2\right)}^2{\left({Z}_{1-\frac{\alpha }{2}}+{Z}_{1-\beta}\right)}^2}{{\left({\overline{x}}_1-{\overline{x}}_2\right)}^2} $$

### Blinding

Researchers were kept blind to groups during data analysis. The dentist conducting exams was blind to condition, design and study purpose. Although participants knew if they had received text messages or not, they were unaware of study design, specific purpose, and hypotheses. Research assistants (RAs) randomzed participants to groups. RAs helping with data collection and cleaning were blind to assigned condition of participants.

### Data analysis

Results were analyzed in SPSS 23.0. The Kolmogorov Smirnov test was employed to determine the normality of data distribution. Analysis of variance (ANOVA) or its non-parametric equivalent was utilized to compare the three groups prior to intervention. Following intervention, to compare attitude, behavioral intention, self-efficacy, practice and plaque of the intervention groups, analysis of covariance (ANCOVA) was used, controlling for baseline level of the dependent variable (e.g., attitude, etc) and demographics. The significance level was set at 0.01.

### Ethics

All participants were informed about the study and confidentiality protocols. Informed consent was obtained from participants. The Ethics Committee of Ahvaz Jundishapur University of Medical Sciences approved the study for research (IR.REFERENCE.REC.1396.554).

## Results

**Fig. 1:**
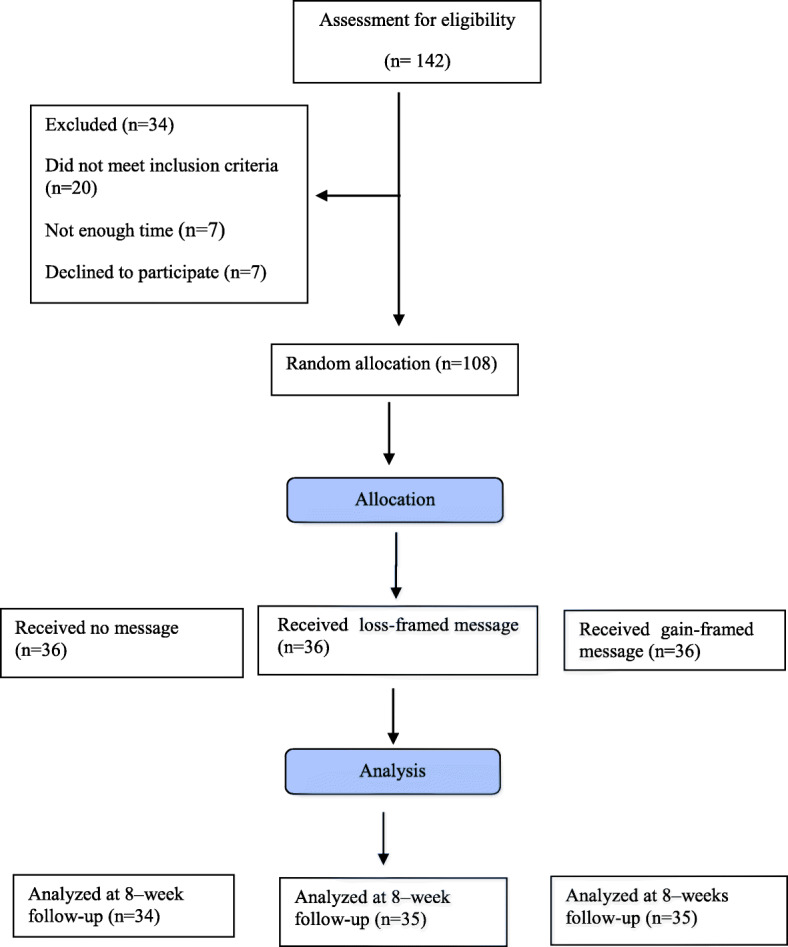
Flow diagram of the participants

Figure 1 shows participant flow through the study. Mean age of participants was M = 27.4 years (standard deviation [SD] = 4.37 years) with a range from 18 to 35 years. Group assignment did not differ by demographic characterstics (e.g., maternal occupation, ethnicity, etc.). See Tables 1 and 2.

**Table 1:** Comparison of quantitative demographic variables

Variable	Gain-framed Mean	Std. Deviation	Loss-framed Mean	Std. Deviation	Control Mean	Std. Deviation	*p*-value*
Maternal age	26.75	4.77	27.69	4.4	27.77	3.94	0.545
Gestational age	22.97	3.71	22.38	3.36	24.33	3.93	0.076

*Derived from ANOVA (analysis of variance). Age provided in years, Std = standard

**Table 2:** Comparison of categorical demographic variables

Variable		Gain; n (%)	Loss; n (%)	Control; n (%)	*p*-value*
Maternal occupation	Employed	2(5.6)	5(13.9)	2(5.6)	0.5
	Housewife	34(94.4)	31(86.1)	34(94.4)	
Maternal level of education	Primary school	3(8.3)	2(5.6)	0	0.463
	Middle school	5(13.9)	3(8.3)	6(16.7)	
	High school	16(44.4)	15(41.7)	12(33.3)	
	University degree	12(33.3)	16(44.4)	18(50)	
Previous pregnancy history	Yes	11(30.6)	14(38.9)	20(55.6)	0.091
	No	25(69.4)	22(61.1)	16(44.4)	
Ethnicity	Lur	33(91.7)	34(94.4)	35(97.2)	0.692
	Arab	2(5.6)	0	0	
	Persian	1(2.8)	2(5.6)	1(2.8)	
Household income	Poor	3(8.3)	9(25)	5(13.9)	0.369
	Moderate	14(38.9)	10(17.8)	14(38.9)	
	Good	19(52.8)	17(47.2)	17(47.2)	
Place of residence	Urban	32(88.9)	35(97.2)	35(97.2)	0.362
	Rural	4(11.1)	1(2.8)	1(2.8)	
Healthcare insurance	Yes	31(86.1)	29(80.6)	29(80.6)	0.775
	No	5(13.9)	7(19.4)	7(19.4)	

*Derived from chi-square, n = number, % = percent

No significant between-groups differences were found at pre-intervention on oral health measures. Table 3 presents M and SD at pre- and post-intervention for plaque and oral health knowledge, attitude, behavioral intention, self-efficacy and practice among intervention and control groups. Normality of groups was examined using the Kolmogrov-Smirnov test and data were found to be normal .

**Table 3:** Oral health knowledge, attitude, intention, efficacy, practice and plaque among groups at baseline and follow-up

Variables				Groups		
	Gain-framed M (SD)	Gain-framed M (SD)	Loss-framed M (SD)	Loss-framed M (SD)	Control M (SD)	Control M (SD)
	Pre intervention	Post intervention	Pre intervention	Post intervention	Pre intervention	Post intervention
Knowledge	5.91(1.65)	11.97(1.33)	6.22(1.57)	11.94(1.10)	5.70(1.71)	6.08(1.79)
Attitude	39.42(4.71)	44.42(3.26)	39.85(4.92)	44.40(3.62)	37.94(5.55)	38.82(4.21)
Behavioral intention	24.37(2.61)	27.28(2.05)	24.51(2.74)	26.77(2.34)	24.05(2.94)	23.67(2.71)
Self-efficacy	34.48(3.84)	38.91(3.23)	34.37(4.20)	38.34(3.42)	34.29(5.24)	33.79(5.15)
Practice	6.02(2.56)	11.00(1.98)	5.51(2.06)	10.25(1.48)	5.94(2.24)	7.55(2.21)
Dental plaque	36.18(14.99)	19.71(9.71)	38.69(15.15)	23.67(10.72)	39.03(12.96)	36.87(11.74)

M = Mean, SD = Standard Deviation. For scoring of scales and possible ranges, see Measures section of Methods

ANCOVA was used to examine post-intervention oral health outcomes across gain−/ loss-framed and control groups, controling for pre-intervention score on each outcome, maternal age/ education and gestational age. Table 4 presents results showing significant differences between groups on each outcome. Following ANCOVA, Tukey’s honestly significant difference (HSD) test was used to determine which particular intervention group means were statistically significantly different from one another.

**Table 4:** Comparison of oral health knowledge, attitude, intention, efficacy, practice and plaque among groups at follow-up

Variables				Groups			*p*-value
	Gain-framed M (SD)	Gain-framed M (SD)	Loss-framed M (SD)	Loss-framed M (SD)	Control M (SD)	Control M (SD)	
	Un-adjusted	Adjusted	Un-adjusted	Adjusted	Un-adjusted	Adjusted	
Knowledge	11.97(1.33)	12.00(7.21)	11.10(4.10)	11.00(5.21)	5.70(1.71)	6.08(1.79)	*p* < 0.001
Attitude	44.30(4.71)	44.00(3.26)	44.30(4.10)	44.00(3.62)	5.55))38.40	39.00(4.21)	*p* < 0.001
Behavioral intention	27.20(2.61)	27.00(2.05)	26.20(2.74)	26.00(2.34)	23.20(2.94)	23.00(2.71)	*p* < 0.001
Self-efficacy	38.03(2.39)	39.42 ± 0	38.34(3.42)	38.00 ± 3.42	33.50(5.24)	33.00(5.15)	*p* < 0.001
Practice	11.00(1.98)	10.00(3.26)	10.25(1.48)	10.00(3.48)	7.20(2.21)	7.00(2.61)	*p* < 0.001
Dental plaque	19.71(9.71)	20.17(9.71)	23.10(10.7)	22.67(10.70)	36.78(11.7)	36.00(5.70)	*p* < 0.001

Note: ANCOVA (analysis of covariance) was used controlling for pre-intervention score on each outcome, maternal age/ education and gestational age. M = Mean, SD = Standard Deviation

Intervention groups had higher knowledge scores compared to the control group (*p* < 0.001), but intervention (gain- vs loss-framed) groups did not differ (*p* = 0.65). For attitude, intervention groups had higher scores than the control group (*p* < 0.001), indicating attitudes conducive to oral health; however, intervention groups were not diferent (*p* = 0.83). For intention and efficacy, intervention groups had higher scores (supporting oral health) than the control group (*p* < 0.001), but intervention groups did not differ on either intention or efficacy (*p* = 0.27 and *p* = 0.78, respectively). For oral health practice, intervention groups had higher mean scores (indicating better oral health practice) compared to control group (*p* < 0.001), whereas no difference was found between gain- and loss-framed intervention groups (*p* = 0.87). There was a significant difference between the mean scores of dental plaque in the intervention and control groups (*p* < 0.001), with intervention groups having less plaque. The gain-framed intervention group had same plaque scores than the loss-framed intervention group (*p* = 0.78).

## Discussion

Using text messaging, the purpose of this study was to investigate the impact of message-framing (gain-framed vs loss-framed vs no message) on oral health-related behaviors and outcomes among pregnant women in Izeh, Iran. The potential of message framing to impact health has been studied with some indication of success [27]. Loss-framed messages might be persuasive for illness detection behaviours, such as X-ray for cavity detection, while gain-framed messages may be more persuasive for illness prevention behaviours, such as tooth-brushing to promote oral health [32–34]. Findings rather consistently showed that both framed messages delivered via text improved dental plaque and oral health knowledge, attitudes, behavioral intentions, efficacy and practices as compared to control condition in which no messages were delivered. However, no differences were found in outcomes for gain- vs loss-framed interventions.

Findings are consistent with work by Ghajari et al. showing that, compared to control condition, both gain- and loss-framed interventions improved student nutrition knowledge [27, 35]. Similarly, messaging-framing has enhanced efficacy for breast-feeding in both gain- and loss-framed interventions, although no difference was found between interventions [26]. Results of the current study are in contrast to a study by Pakpour et al. in which loss-frame messages related to toothbrusing and flossing as compared to gain-framed messages [36, 37]. Similarly, Gallagher et al. found that, as compared to loss-framed messages, gain-framed messages better related to preventive health behaviors (e.g., skin cancer, exercise, smoking cessation [32];). These disparate findings may relate to as yet unidentified moderating factors [27], including cultural factors [36, 38]. Based on the Theory of Motivation, individuals who are approach-oriented may change behavior through gain-framed messages and those who are avoidance-oreinted may be more responsive to loss-framed messages [39]. Therefore, future studies may wish to examine such moderating factors that could influence outcomes.

Use of cell phones can have a significant impact on self-regulation as well as attitudes towards cell phone-assisted learning [40]. Use of texting to deliver health interventions can eliminate service barriers, including transportation, travel time, and some factors related to rapport [26, 41]. Use of mobile phones to deliver an intervention to patients with a blood disorder improved self-care knowledge, attitudes and behaviors as compared to control group [41]. Similarly, use of text-messaging to provide antenatal education was found to be effective [42]. Although the present study did not find differential support for gain- vs loss-framed messages, the texting intervention was effective in impacting oral health in pregnant women.

### Limitations

This study recruited women receiving pregnancy care at a health clinic. It is possible that results may not apply to women unable to attend a health clinic. Similarly, women in the study volunteered to participate, and therefore may be unusually motivated to engage in healthcare interventions. Future studies may wish to proactively seek pregnant women not engaged in pregnancy care at a clinic. Women in the control condition received no texts. Future studies may wish to control for receiving generic advice on oral health via text. Finally, follow-up took place 8 weeks following end of texting. Longer term follow-up should be considered in future work.

## Conclusion

As compared to receiving no messages, text message interventions using gain- and loss-framed messages improved dental plaque and oral health knowledge, attitude, behavioral intention, self-efficacy and practices among pregnant women. Framing did not have an impact on outcome.

## Supplementary Information

**Additional file 1.** The questionnire used in the study to collect the data. The first part of the questionnaire included demographic characteristics as well as previous pregnancy history which was comprised of 14 items. The second part of the questionnaire consisted of oral health knowledge, attitude, behavioral intention, self-efficacy, and practice.

**Additional file 2.** Messages sent. 30 gain-framed and 30 loss-framed messages were sent to the respective conditions (i.e., gain- or loss-framed).

## Data Availability

Upon request, onsite (Ahvaz Jundishapur University of Medical Sciences, Ahvaz, Iran) access to the data can be provided.

## Abbreviation

NPI: Navy Plaque Index
