# Supplementary material for: Effectiveness of message-framing to improve oral health behaviors and dental plaque among pregnant women
Source: Arch Public Health. 2021 Jun 28;79:117. doi: 10.1186/s13690-021-00640-1 (PMC8237427; doi:10.1186/s13690-021-00640-1)
Supplement: Supplementary file 1 — Additional file 1. The questionnire used in the study to collect the data. The first part of the questionnaire included demographic characteristics as well as previous pregnancy history which was comprised of 14 items. The second part of the questionnaire consisted of oral health knowledge, attitude, behavioral intention, self-efficacy, and practice. [file 13690_2021_640_MOESM1_ESM.doc]

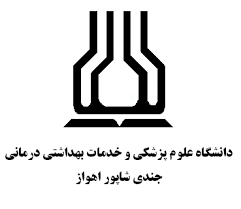


Ahvaz Jundishapur University of Medical Sciences

School of Public Health

Department of Public Health and Promotion

| **Dear Mother**  You are highly appreciated for your cooperation and the time devoted to this study. You are kindly requested to carefully review the following questionnaire. It has been developed to understand oral health of mothers. There is no need to write your name and surname. Your information will be kept confidential.  **Thank You**  **Demographic information:**  **1. Mother’s age: 2. Husband’s age: 3. Years of marriage: 4. Pregnancy week:**  **5. Mother’s occupation: employed 🞎 housewife 🞎**  **6. Husband’s occupation: employed 🞎 worker 🞎 self-employment🞎 unemployed 🞎**  **7. Medical insurance coverage: Yes 🞎 No 🞎**  **8. Mother’s education: primary 🞎 middle 🞎 high school 🞎 university 🞎**  **9. Husband’s education: primary 🞎 middle 🞎 high school 🞎 university 🞎**  **10. Previous pregnancy: Yes 🞎 No 🞎**  **11. Mother’s ethnicity: Lur 🞎 Kurd 🞎 Turk 🞎 Arab 🞎 Persian 🞎**  **12. How do you describe your income?**  **1) Low (unable to cover living expenses) 2) Average (lower than upper bound of living expenses)**  **3) Good (provides for living expenses) 4) Excellent (higher than what’s needed for living expenses)**  **13. Number of family members: …………………..**  **14. Place of residence: City 🞎 Village 🞎**  **15. Percentage of dental plaque: ………………….. (completed by professional)** |
| --- |

| **Mothers’ awareness of oral and dental care during pregnancy** | **Code** |
| --- | --- |
| Using which one of the following increases the tooth decay?  a) Sweet and sticky ingredients b) Fruits and vegetables c) Dairy d) All of the above | **1** |
| When is the best time during pregnancy to see the dentist to address tooth decay?  a) First three months b) Second three months c) Third three months d) All of the above | **2** |
| How often per day should teeth be brushed?  a) 1 b) 2 c) 3 d) 4 | **3** |
| Of the below choices, which is best to enhance oral health?  a) Toothbrush and dental floss b) Mouthwash c) Chewing gum d) Fresheners | **4** |
| Frequent vomiting during pregnancy leads to which kind of initial damage?  a) Tooth abrasion b) Toothache c) Gum disease d) Dental plaque | **5** |
| Of the below choices, which is the worse choice for times per day to brush teeth?  a) 2 b) 3 c) 4 d) 5 | **6** |
| Which of the following disease is (diseases are) related to oral health?  a) Diabetes b) Cardiovascular disease c) Cancers d) All of the above | **7** |
| When is the most important time for brushing/ flossing teeth?  a) After sleep b) Before sleep c) After breakfast d) After each main meal | **8** |
| The first visit to the dentist should be made during which month of pregnancy?  a) 1^st^ month b) 2^nd^ month c) 3^rd^ month d) 4^th^ month | **9** |
| At least how many visits to the dentist are necessary during pregnancy?  a) 1 b) 2 c) 3 d) 4 | **10** |
| A toothbrush should be replaced after how many months of use?  a) 1 b) 3 c) 6 d) 8 | **11** |
| A person with no oral health problem should visit the dentist how often? Every…  a) 3 months b) 6 months c) 1 year d) 2 years | **12** |
| Which is the best tool for cleaning the space between teeth?  a) Toothbrush b) Interspace brush c) Dental floss d) Toothpick | **13** |
| For how long should mouthwash be kept in the mouth?  a) 30 sec. to 1 min. b) 1 min. c) 1-2 min. d) 2-4 min. | **14** |

| **Attitude questions** | |
| --- | --- |
| If I don’t take care of my teeth, I will lose them.  Strongly agree 🞎 Agree 🞎 No opinion 🞎 Disagree 🞎 Strongly disagree 🞎 | **1** |
| If I lose my teeth, I will be ugly while smiling.  Strongly agree 🞎 Agree 🞎 No opinion 🞎 Disagree 🞎 Strongly disagree 🞎 | **2** |
| Using a toothbrush during pregnancy is boring.  Strongly agree 🞎 Agree 🞎 No opinion 🞎 Disagree 🞎 Strongly disagree 🞎 | **3** |
| Using dental floss during the pregnancy is boring.  Strongly agree 🞎 Agree 🞎 No opinion 🞎 Disagree 🞎 Strongly disagree 🞎 | **4** |
| Dental services during pregnancy are dangerous for fetal health.  Strongly agree 🞎 Agree 🞎 No opinion 🞎 Disagree 🞎 Strongly disagree 🞎 | **5** |
| When my teeth are healthy, it is a waste of time to visit the dentist.  Strongly agree 🞎 Agree 🞎 No opinion 🞎 Disagree 🞎 Strongly disagree 🞎 | **6** |
| I believe that a pregnant woman’s teeth are more prone to decay than other people.  Strongly agree 🞎 Agree 🞎 No opinion 🞎 Disagree 🞎 Strongly disagree 🞎 | **7** |
| Due to fear of nausea, I don’t use a toothbrush or dental floss.  Strongly agree 🞎 Agree 🞎 No opinion 🞎 Disagree 🞎 Strongly disagree 🞎 | **8** |
| I fear dental services could negatively impact my fetus, so I don’t visit the dentist.  Strongly agree 🞎 Agree 🞎 No opinion 🞎 Disagree 🞎 Strongly disagree 🞎 | **9** |
| Due to the impact of oral health on my fetus, I use a toothbrush and dental floss.  Strongly agree 🞎 Agree 🞎 No opinion 🞎 Disagree 🞎 Strongly disagree 🞎 | **10** |

| **Behavioral intention questions** | |
| --- | --- |
| I would like to visit the dentist during my pregnancy.  Strongly agree 🞎 Agree 🞎 No opinion 🞎 Disagree 🞎 Strongly disagree 🞎 | **1** |
| To prevent oral disease, I want to use a toothbrush regularly during pregnancy.  Strongly agree 🞎 Agree 🞎 No opinion 🞎 Disagree 🞎 Strongly disagree 🞎 | **2** |
| To prevent oral disease, I will use dental floss regularly during pregnancy.  Strongly agree 🞎 Agree 🞎 No opinion 🞎 Disagree 🞎 Strongly disagree 🞎 | **3** |
| To learn how to use a toothbrush and dental floss properly, I want to consult with a health specialist at my healthcare center.  Strongly agree 🞎 Agree 🞎 No opinion 🞎 Disagree 🞎 Strongly disagree 🞎 | **4** |
| If I need any dental services during pregnancy, I will seek them immediately.  Strongly agree 🞎 Agree 🞎 No opinion 🞎 Disagree 🞎 Strongly disagree 🞎 | **5** |
| To prevent tooth decay, I want to use less sugar during pregnancy.  Strongly agree 🞎 Agree 🞎 No opinion 🞎 Disagree 🞎 Strongly disagree 🞎 | **6** |

| **Mothers’ self-efficacy for oral health** | |
| --- | --- |
| If I don’t know how to brush my teeth properly, I can learn.  Strongly agree 🞎 Agree 🞎 No opinion 🞎 Disagree 🞎 Strongly disagree 🞎 | **1** |
| To have healthy teeth, I can eat less chocolate and sugars.  Strongly agree 🞎 Agree 🞎 No opinion 🞎 Disagree 🞎 Strongly disagree 🞎 | **2** |
| I can use dental floss regularly during pregnancy.  Strongly agree 🞎 Agree 🞎 No opinion 🞎 Disagree 🞎 Strongly disagree 🞎 | **3** |
| During pregnancy, I can visit the dentist regularly.  Strongly agree 🞎 Agree 🞎 No opinion 🞎 Disagree 🞎 Strongly disagree 🞎 | **4** |
| During pregnancy, I can regularly brush my teeth.  Strongly agree 🞎 Agree 🞎 No opinion 🞎 Disagree 🞎 Strongly disagree 🞎 | **5** |
| I can find enough time for brushing my teeth or using dental floss.  Strongly agree 🞎 Agree 🞎 No opinion 🞎 Disagree 🞎 Strongly disagree 🞎 | **6** |
| Despite my business, I can visit the dentist regularly.  Strongly agree 🞎 Agree 🞎 No opinion 🞎 Disagree 🞎 Strongly disagree 🞎 | **7** |
| If I have morning sickness, I can wash my mouth after each time I vomit.  Strongly agree 🞎 Agree 🞎 No opinion 🞎 Disagree 🞎 Strongly disagree 🞎 | **8** |
| Even if I fear dental services, I can engage in them regularly.  Strongly agree 🞎 Agree 🞎 No opinion 🞎 Disagree 🞎 Strongly disagree 🞎 | **9** |

| **Mothers’ performance on oral and dental healthcare** | |
| --- | --- |
| How often do you brush your teeth every day?  a) Never b) 1 c) 2 d) 3 e) More than 3 times | **1** |
| How often do you usually use dental floss every day?  a) Never b) 1 c) 2 d) 3 e) More than 3 times | **2** |
| What time of day do you brush your teeth the most?  a) Morning b) Afternoon c) Evening d) After eating sweets e) Never | **3** |
| Have you ever regularly had an oral health examination by a healthcare professional?  Yes 🞎 No 🞎 | **4** |
| How often do you visit to dentist? Every…  a) 2 months b) 4 months c) 6 months d) One year or more e) Never | **5** |
| How to brush teeth properly (observing with model). Completed by staff.  a) Correct b) To some extent correct c) Wrong | **6** |
| How to use dental floss properly (observing with model). Completed by staff.  a) Correct b) To some extent correct c) Wrong | **7** |
| Do you use mouthwash? Yes 🞎 No 🞎 | **8** |
| Before pregnancy, had you visited to the dentist? Yes 🞎 No 🞎 | **9** |
| Do you brush your teeth immediately after eating sweets? Yes 🞎 No 🞎 | **10** |
| Do you wash your mouth after vomiting? Yes 🞎 No 🞎 | **11** |
| Which was your last visit to the dentist?  a) I’ve never visited. b) Less than 3 months ago c) 4-6 months ago  d) 7-12 months ago e) More than one year ago | **12** |
| Do you use toothpaste with fluoride? Yes 🞎 No 🞎 | **13** |
| Do you brush your teeth before going to sleep? Yes 🞎 No 🞎 | **14** |
