# Supplementary material for: Effectiveness of message-framing to improve oral health behaviors and dental plaque among pregnant women
Source: Arch Public Health. 2021 Jun 28;79:117. doi: 10.1186/s13690-021-00640-1 (PMC8237427; doi:10.1186/s13690-021-00640-1)
Supplement: Supplementary file 2 — Additional file 2. Messages sent. 30 gain-framed and 30 loss-framed messages were sent to the respective conditions (i.e., gain- or loss-framed). [file 13690_2021_640_MOESM2_ESM.docx]

**Framed Health Messages**

| **No.** | **GAIN-Framed Message** | **LOSS-Framed Message** |
| --- | --- | --- |
| 1 | Maintaining proper oral hygiene can prevent gum disease during pregnancy. | Lack of proper oral hygiene exacerbates gum disease during pregnancy. |
| 2 | Proper use of fluoride mouthwash will definitely, and to a large extent, prevent the development and progression of cavities. | Failure to use fluoride mouthwash can lead to cavities. |
| 3 | By maintaining oral hygiene, permanent teeth are kept for a lifetime | When oral hygiene is not properly maintained, permanent teeth are more likely to decay. |
| 4 | To prevent oral disease, teeth should be brushed at least twice daily: In the morning, before or after breakfast; and at night, before going to bed. | If tooth-brushing is not performed at least twice daily, in the morning before or after breakfast and at night before going to bed, oral disease occurs. |
| 5 | Changing a toothbrush every 3 months reduces the possibility of contamination and oral disease by toothbrushes. | Keeping a toothbrush for more than 3 months increases the possibility of contamination and oral disease by a toothbrush. |
| 6 | Flossing should be done at least before bedtime to prevent tooth decay. | If surfaces between teeth are not cleaned with dental floss at least once a day before going to bed, teeth will decay. |
| 7 | Individuals who brush and floss properly on a daily basis will have healthier teeth. | Individuals who do not correctly brush and floss daily will suffer from tooth decay. |
| 8 | Those who brush and floss properly on a daily basis will not suffer from bad breath. | Those who do not brush and floss properly on a daily basis will suffer from bad breath. |
| 9 | Did you know using fluoride mouthwash once a week can help prevent tooth decay? | If you do not use fluoride mouthwash once a week, you are more likely to have tooth decay. |
| 10 | If you see a dentist regularly every 6 months, your oral health problems will be diagnosed sooner. | If you do not see to a dentist regularly every 6 months, your oral health problems will go undetected for longer and problems will become more severe. |
| 11 | Timely diagnosis and treatment of tooth decay by a dentist will reduce the effects of cavities and oral health problems. | When tooth decay is not diagnosed and treated in time, cavities will progress and cause various oral health problems. |
| 12 | Consuming foods with less sugar reduces your risk of oral disease. | Consuming foods with more sugar increases your risk of oral disease. |
| 13 | Flossing after each meal will help reduce cavities to a great extent. | If you do not use dental floss, you have a greater chance of cavities. |
| 14 | Keeping mouth wash in your mouth for 30-60 seconds helps ensure the health of your teeth. | Keeping mouthwash in your mouth for less than 30-60 seconds reduces its effect to keep your teeth healthy. |
| 15 | If you brush your teeth immediately after consuming sweets, your tooth decay will be reduced. | If you do not brush your teeth immediately after consuming sweets, your tooth decay will increase. |
| 16 | Early diagnosis of infection and inflammation of gums, teeth, and oral problems in the 2^nd^ trimester helps the health of mother and fetus. | It is necessary to see a dentist in the 2^nd^ trimester because not treating infection and inflammation of gums, teeth, and oral problems can be dangerous for the health of mother and fetus. |
| 17 | By brushing your teeth for at least 4 min, your teeth will get cleaner and healthier. | Brushing your teeth for less than 4 min, will not be effective in promoting oral health. |
| 18 | Pregnant women should reduce their risk of cavities by brushing at least twice a day with fluoride toothpaste. | Brushing less than at least twice a day increases the risk of cavities. |
| 19 | Proper dental care during pregnancy can reduce oral health problems in newborns and reduce tooth decay in infants. | Improper dental care during pregnancy increases the likelihood of oral health problems in newborns and tooth decay in infants. |
| 20 | Pregnant women should rinse their mouths immediately after eating food high in carbohydrates (for example, rice, sweets and bread), or consume such food less frequently. | Pregnant women should rinse their mouths immediately after eating food high in carbohydrates (for example, rice, sweets and bread), or they are at increased risk for tooth decay. |
| 21 | Oral healthcare during pregnancy is very important because it enhances the health of both you and your baby. | Lack of oral healthcare during pregnancy can endanger the health of you and your baby. |
| 22 | By reducing the consumption of sweet foods during pregnancy, dental plaque and other oral health problems are reduced. | Sweet foods lead to plaque formation on teeth, which can lead to tooth decay and cause oral health problems. |
| 23 | Each time you vomit during pregnancy, rinse your mouth with water to reduce tooth decay caused by stomach acid. | If you vomit during pregnancy and don’t rinse your mouth, stomach acid can cause severe tooth decay. |
| 24 | By consuming fruits, vegetables and dairy products during pregnancy, your tooth decay will be reduced. | If you do not eat fruits, vegetables and dairy products during pregnancy, your tooth decay will increase. |
| 25 | Tooth decay can be prevented during pregnancy by following a healthy diet and oral hygiene. | The increase in cavities during pregnancy is mainly due to poor diet and oral hygiene. |
| 26 | Using fluoride mouthwash once a week can protect worn or sensitive teeth. | Failure to use fluoride mouthwash once a week will expose your worn or sensitive teeth to more decay. |
| 27 | Bacteria that cause oral health problems can be transmitted to the fetus; therefore, your baby is less likely to develop cavities in the future if you have good oral hygiene during pregnancy. | Bacteria that cause oral health problems can be transmitted to the fetus; therefore, failure to use good oral hygiene during pregnancy increases the chances that your baby will have cavities in the future. |
| 28 | Pregnant women who use a toothbrush and floss properly clean plaque from their teeth, which reduces oral health problems. | Pregnant women who do not use a toothbrush and floss properly do not clean plaque from their teeth, which worsens oral health problems. |
| 29 | Proper oral health is helpful in the reduction of cardiovascular diseases, type 2 diabetes, and cancer risks. | Oral disease is associated with cardiovascular diseases, type 2 diabetes, and cancer. |
| 30 | Oral hygiene in pregnant women is effective in reducing the risk of preterm delivery. | The lack of oral hygiene in pregnant women increases the risk of preterm delivery by 7 fold. |

No. = Number.
